# The Effects of Foreknowledge and Task-Set Shifting as Mirrored in Cue- and Target-Locked Event-Related Potentials

**DOI:** 10.1371/journal.pone.0049486

**Published:** 2012-11-12

**Authors:** Mareike Finke, Carles Escera, Francisco Barceló

**Affiliations:** 1 Institute for Brain, Cognition and Behavior (IR3C), University of Barcelona, Barcelona, Spain; 2 Cognitive Neuroscience Research Group, Department of Psychiatry and Clinical Psychobiology, University of Barcelona, Barcelona, Spain; 3 Laboratory of Clinical Neuropsychology, Department of Psychology, University of the Balearic Islands, Palma de Mallorca, Spain; Bellvitge Biomedical Research Institute-IDIBELL, Spain

## Abstract

The present study examined the use of foreknowledge in a task-cueing protocol while manipulating sensory updating and executive control in both, informatively and non-informatively pre-cued trials. Foreknowledge, sensory updating (cue switch effects) and task-switching were orthogonally manipulated in order to address the question of whether, and to which extent, the sensory processing of cue changes can partly or totally explain the final task switch costs. Participants responded faster when they could prepare for the upcoming task and if no task-set updating was necessary. Sensory cue switches influenced cue-locked ERPs only when they contained conceptual information about the upcoming task: frontal P2 amplitudes were modulated by task-relevant cue changes, mid-parietal P3 amplitudes by the anticipatory updating of stimulus-response mappings, and P3 peak latencies were modulated by task switching. Task preparation was advantageous for efficient stimulus-response re-mapping at target-onset as mirrored in target N2 amplitudes. However, N2 peak latencies indicate that this process is faster for all repeat trials. The results provide evidence to support a very fast detection of task-relevance in sensory (cue) changes and argue against the view of task repetition benefits as secondary to purely perceptual repetition priming. Advanced preparation may have a stronger influence on behavioral performance and target-locked brain activity than the local effect of repeating or switching the task-set in the current trial.

## Introduction

The brain’s ability to represent, maintain and update contextual (*task-set*) information enables us to alternate successfully between tasks [1], [2]. Task-set reconfiguration is required when task demands change, as goal directed behavior needs to be adjusted to the new task. Task-cueing paradigms are used to investigate the underlying processes such as attentional shifting from one task to the other (task-switching), retrieval of goals and rules, and the activation of the current task-set, or the inhibition of the previous irrelevant one [3], [4].

Task-switching effects are inversely proportional to the length of the preparation interval. The so-called task-switch cost is the additional time needed to switch a task compared to a task repetition. However, a residual switch cost always remains, suggesting that anticipatory preparation cannot fully overcome the cost of switching tasks [5]–[7]. Anticipatory effects and goal activation have been explored by manipulating the preparation time and the information content conveyed at cue onset [4], [8]–[11]. In spite of a wide consensus that task preparation is advantageous, the underlying cerebral processes are still a matter of debate. There is evidence about the relative independence of a mechanism of general task preparation (independent from the upcoming task), and specific task activation (i.e., either repeat the former task or switch to another task). This has been tested in different studies that manipulated the informational content of the cue. In informatively cued trials the cue contained specific task-relevant information, i.e., the stimulus-response (S-R) rule for the upcoming task, while this was not the case in non-informatively cued trials. The two processes of task preparation and specific goal activation engage common and distinct areas of prefrontal cortex, as activating Brodmann areas (BA) 45, 46 and 40 varies depending on foreknowledge, while activation in BA 8, 39 and 40 is modulated by task switching [9]. Moreover, there is evidence that an early event-related potential (ERP) positivity reflects the differences between informative and non-informative cues while differences between task repeat and task switch occur later in time [4]. This is consistent with the idea of various independent processes in task-switching [4], [5], [9], [12]. A related and important ongoing debate in the task-switching literature is whether, and to which extent, task switch costs can be attributed to a sensory change in the task-indicating cue [13]–[18]. Most previous studies that researched the effects of a cue change on the final switch cost employed informative cues with a 2∶1 cue-task mapping, and hence, cue switches and cue repetitions both conveyed relevant information about the upcoming task. These studies suggested that cue repetition benefits could partly explain the switch costs (cf., [13], [14]). However, these studies did not clarify whether such cue repetition benefits (or cue switch costs) would also be found for non-informative cues that do not convey any information about the upcoming task. By using two sensory different non-informative cues, we investigated whether task-irrelevant sensory changes, which are unrelated to any task rule, can also modulate behavior and target-locked ERPs in a similar way as informative cue changes do [13], [18].

Additionally to the question of whether the cue repetition effect is (partially) related to sensory mechanisms, the current design enables us to investigate how non-informative cue switches also modulate target-locked ERPs. Up to date cue switch effects have been investigated using informative cues only [18], [19]. However, it remains to be known whether and how a sensory switch in a non-informative cue can bias the ensuing target-locked ERPs. The manipulation of a Cue type factor (informative versus non-informative) and a Task condition factor (including cue repeat, cue switch and task switch) allowed us to examine to which extent sensory updating and task-switching differentially contribute to the behavioral and brain responses with and without foreknowledge about the upcoming task. Thus, for instance, a cue switch in a non-informatively cued trial might increase alertness and speed up the response to the upcoming target in a task non-specific manner [20]. In such a case, we would hypothesize an interaction between the Cue type and Task condition factors on target-locked brain activity. On the contrary, if non-informatively cued switches influenced processing at a strictly sensory level, then target-locked brain activity should not be modulated by the interaction of the Cue type and Task condition factors. Several previous studies have shown that the mean amplitude of the cue-locked P3 component in the ERPs are modulated by both sensory updating and task switching, at least in the auditory modality [15], [19], [21]. Even though less well studied than the P3, the endogenous and fronto-centrally distributed P2 component has also been shown to be sensitive to change detection [22], [23]. Some authors have proposed that this fronto-central P2 component reflects the detection of stimulus salience and stimulus evaluation processes [24], and recent studies showed that this component is modulated by preparatory attentional control [11], [25]–[27]. According to the existing evidence, we hypothesized that mean cue-locked P2 amplitudes at fronto-central scalp regions will be modulated by task switching, but might also by the type of cue-information content (foreknowledge). With the present task design we want to answer the question whether the P2 is a general “change detector” or rather a more specific “*task-related* change detector” which would not be affected by task-irrelevant changes. Consequently, we want to light up further the interpretation of previous P2 effects found in response to task-switching cues, and whether these P2 effects could be related either to the early (sensory) processing of the cue, or to the complexity of the upcoming task [22], [23], [27]. Under the first hypothesis, cue-locked P2 should be enhanced both in cue and task switch trials, whereas from the latter interpretation the cue-locked P2 should be significantly enhanced in task switch trials only. Regarding the P3 we expect different modulations depending on the task and the foreknowledge as former work could link this component to working memory and cognitive control processes [15], [19]. Enhanced P3 amplitudes should appear for informatively cued trials compared to non-informatively cued trials mirroring the general processes of cue-response mapping and anticipatory task preparation. Moreover, P3 amplitudes are expected to differ depending on task condition as we assume that the process of reloading the previous task-set into working memory should lead to smaller P3 amplitudes compared to an updating of cue-response mapping, as only the latter includes a task-set reconfiguration process [28]–[30]. In the present study we expect similar effects for the visual modality, although smaller P3 amplitudes are often observed in response to visual stimuli as compared to auditory stimuli. Apart from our interest in the distinct processes related to cue encoding in task-switching, the study aimed to elucidate how general or specific anticipatory task preparation influence target-locked brain activity. Former studies found a clear task switch effect in target-locked brain activity, but only for informative trials [4]. Moreover, there is evidence that task preparation diminishes task switch effects but cannot override it entirely as visible in ERP modulations such as P3, late positive potentials and the switch negativity [4], [6], [15], [17], [19], [21]. The target-locked N2 is thought to indicate action monitoring as well as target-selection and response preparation [4], [28], [31]–[34]. Regarding task-switching, the target N2 has been related to post-perceptual and executive processes such as intentional task-set reconfiguration and other aspects of task switching [32]. Thus, task-preparation and task implementation both modulated the target N2 in a recent study [28]. In the present study we manipulated task-preparation and task implementation by using informative and non-informative cues, and three different task conditions, respectively. The N2 component seems to be a promising component to look at both processes in order to find out how each of them leads to modulations in the target-locked ERPs or whether cue and task condition might interact. We expect target-locked N2 to be modulated by both cue and task. Moreover, the possibility to explore the interaction between these factors will allow us to shed new light on how non-informative cue switches also affect the target-locked N2. Finally, we predicted prolonged response times for all non-informatively cued trials, as well as for cue switch and task switch trials compared to task repeat trials in the informatively cued condition [4], [9], [21].

## Materials and Methods

### Participants

Seventeen healthy individuals (3 male, mean age 23.5 years ±0.92 [SEM], range 19–33 years) recruited from the University of Barcelona participated in the study. All participants were right-handed and had normal or corrected-to-normal vision. None of the subjects reported a neurological or psychiatric history.

### Ethics Statement

Participants gave informed written consent before the experiment. The experimental protocol was approved by the Ethical Committee of the University of Barcelona and was in accordance with the Code of Ethics of the World Medical Association (Declaration of Helsinki).

### Task and Procedure

**Figure 1:**
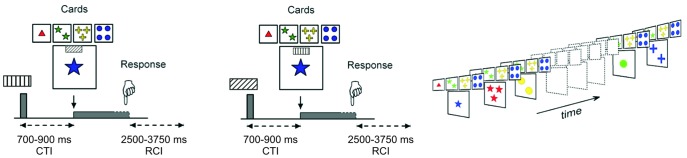
Stimulus material and experimental design. Each trial consisted on a visual cue, either informative (Ic) or non-informative (NIc), followed by the target cards to be sorted. Target cards also contained a similar cue (this was a non-informative cue in informatively cued trials, and vice versa). Participants had to match the choice card with one of the four key cards according to either the color or shape of their elements. Examples of an Ic trial (left panel), and a NIc trial (middle panel) are shown on the figure.

A computerized task-cueing protocol inspired by the original Wisconsin Card Sorting Test and adapted for measuring ERPs was used [35]. Each trial consisted of a visual cue followed by a target display with four key cards on the top of one choice card that had to be matched with one of the key cards either by color or shape (Figure 1). Stimuli were presented centrally on a computer screen with display subtending a visual angle of 6° horizontally and 5° vertically. Stimuli remained on the screen until a response was given. Response times (RT) and hit rates were recorded using Presentation® (Neurobehavioral Systems, Inc). Each trial began with the presentation of a visual explicit cue that could be either informative (Ic) or non-informative (NIc). In total, six differently striped rectangles were used (white frame and white stripes on a black background). The two sorting rules, color and shape, were indicated by two different rectangles per rule to disentangle the effects of cue and task switching. The actual rule could be read out via the combination of rectangle orientation (vertical/horizontal) and line orientation (vertical/horizontal) as shown in Figure 1. For instance, the color rule was indicated by a horizontal rectangle with vertical stripes, and also by a vertically oriented rectangle with horizontal stripes. The shape rule was indicated by a horizontal rectangle with horizontal stripes, and also by a vertical rectangle with vertical stripes. Two diagonally striped rectangles, one horizontal, one vertical, did not supply any information about the ongoing sorting rule. Instead, in non-informatively cued trials, the contextual information about the ongoing sorting rule was presented simultaneously with target onset. In order to keep the physical similarity between the two cueing conditions, a non-informative cue was presented in the target period of informatively cued trials. Consequently, the participants were required to use informative cues in order to prepare for the upcoming task, as no rule information was present at the target period. In turn, no preparation was possible in the non-informatively cued trials. Noteworthy, participants always received information about the ongoing sorting rule in every trial, either at cue onset or at target onset, and hence, every trial contained information about how to sort the cards. Before starting with the task, the cue mapping was explained to the participants and they were informed that the correct rule would change unpredictably after a variable number of card sorts, and that they would have to shift the sorting rule consequently. Each participant completed a practice block before starting the experimental session, to make sure that they understood the task instructions.

Three task conditions were defined to dissociate the effects of cue- and task-switching in both informatively and non-informatively cued trials. Firstly, there were repeat trials where both cue and task were repeated relative to the previous trial (cue repeat). Secondly, there were cue-switch trials, where only the cue changed but the task repeated compared to the previous trial (cue switch). Finally, in task-switch trials both cue and task changed (task switch). This design allowed us an orthogonal manipulation of cue switches involving either a sensory change only, or a change in both sensory and higher-order task-set information. Ideally, a combination of cue-repeat and task-switch conditions would be possible in non-informative trials. This condition has been used in former studies that explored the influence of independent changes in cue and task on task performance [13], [16], [17]. However, such a combination cannot be implemented for informative trials, and hence, it will not be considered in this study. The present 2∶1 mapping between cues and task rules was meant as a control for cue switch costs as opposed to task switch costs [13], as it has been recently shown to modulate ERPs differently [17], [21]. Informative and non-informative cues occurred with the same overall probability each over the course of the experiment. Additionally, all three task conditions appeared equiprobably (33.33% each), and so were all possible cue-task combinations (16.67% each). After a practice block, each participant performed 312 trials grouped into six blocks within which changes in cue and task occurred in a semi-randomized order. The cue-target interval (CTI) varied randomly between 700 and 900 ms to minimize the effects of a constant preparation interval. The target remained on the screen until a response was given. The response-to-cue interval (RCI) also varied randomly between 2500 and 2750 ms to prevent systematical noise in the cue-locked ERPs. The jitter in both CTIs and RCIs was meant to minimize the effects of time estimation processes -as distinct from anticipatory task-set preparation- in the pre-stimulus interval.

Participants used their index and middle fingers of both hands to match the choice card with one of the four key cards. The far left button designated the key card on the far left of the display and the far right button designated the card on the far right and so on. The task sets as described above consisted of a 4-stimulus to 4-response mapping, and participants used their left/right hand for the two left/right buttons, respectively. For instance, when sorting by the “shape” rule, a triangle choice card was to be matched with the triangle key card by using the left-most button on the response panel (Figure 1).

### EEG Recording

The EEG was recorded from 61 scalp electrodes positioned according to the extended 10–20 system. The reference electrode was placed on the tip of the nose. Horizontal and vertical electro-oculographic recordings (EOG) were recorded with electrodes placed below and at the outer canthi of the right eye. The EEG was amplified and digitized at 512 Hz and impedances were kept below 10 kΩ during the whole recording session. EEG data was processed offline with a band pass filter from 0.5–40 Hz. EOG correction was performed by applying the blind source separation technique with ASA 4.7.3 of ANT® Software (Enschede, The Netherlands), as described in Belouchrani and colleagues [36]. After EOG correction, any epochs containing EEG activity exceeding ±75 µV were rejected from further analysis. This procedure resulted in a final rejection of 4.71% of all correct trials. Mean amplitudes of selected cue- and target-locked visual ERP components were computed over a time window of 800 ms including a 100 ms pre-stimulus baseline.

### Data Analysis

For behavioral analysis, a correct trial was defined as a correct button press occurring between 100 and 3000 ms from target onset. Mean RT relative to target-onset was computed for correct trials only. RT and hit rate were analyzed using repeated measures 2×3 ANOVAs with two within-subject factors: Cue type (informative, non-informative) and Task condition (cue repeat, cue switch, task switch).

Our choice of analysis windows and channels was done according to former task-switching studies (cf., [4], [19], [21], [25], [37]), and in agreement with a visual inspection of mean ERP data. Mean amplitudes were extracted for the cue-locked P2 in the window of 180–220 ms at F1, Fz, F2, FC1, FCz, FC2 as in West et al., 2011 [23]. A similar analysis windows for mean P2 amplitudes were used in previous studies [19], [25], [37]. Mean cue-locked P3 amplitudes (450–550 ms; CP1, CPz, CP2, P1, Pz, P2) were measured within the same time window as Jamadar and colleagues (2010; also cf., [19], [25]). Target-locked N2 peak amplitude and latency were measured at C1, Cz and C2 sites in the time window 200–500 ms, following former literature which researched modulations in the N2 regarding cognitive control [29], [37]–[39]. Target-locked N2 analyses were performed at mid-central sites, consistent with previous studies which described the central N2 peaking at Cz [40], and with the scalp topographies of target-locked peak N2 amplitudes for both informatively cued trials and non-informatively cued trials. One study that guided our choice of this rather wide window for peak N2 latency is by Leleu and colleagues (2010) [31]. These authors analyzed the N2 component within a 400 ms time window (200–600 ms) and found task related effects from 200–450 ms post-target.

For the analysis of target-locked ERPs, a full factorial design was used including Cue type (informative, non-informative) and Task condition (cue-repeat, cue-switch, and task-switch) and Laterality (left, central, right).

In order to examine whether a pure sensory change in cue modulates performance and brain activity, we compared cue repeat trials with cue switch trials for both, informative and non-informative trials using repeated ANOVAs with a 2 (Cue type) × 2 (Task condition) × 2 (Frontality) × 3 (Laterality) design. Due to the null result of this analysis, a random selection of trials assured similar numbers of switch and repeat trials across both non-informatively and informatively cued trials. Moreover, data was subsequently subjected to a second repeated measures ANOVA with factors: 3 (Trial type: non-informative cued trials, informative task repeat, informative task switch) × 2 (Frontality) × 3 (Laterality). Channel lines F and CP served as Frontality level 1 and lines FC and P as factor level 2, respectively for the analysis of the P2 and P3. The three levels for Laterality are associated with the left, central and right channels. All post-hoc tests for behavioral and ERP analyses were performed with t-tests and a Bonferroni correction was used to adjust p-values for all multiple pairwise contrasts. Greenhouse-Geisser corrections were used to adjust degrees of freedom whenever the assumption of sphericity was violated.

## Results

### Performance

**Figure 2:**
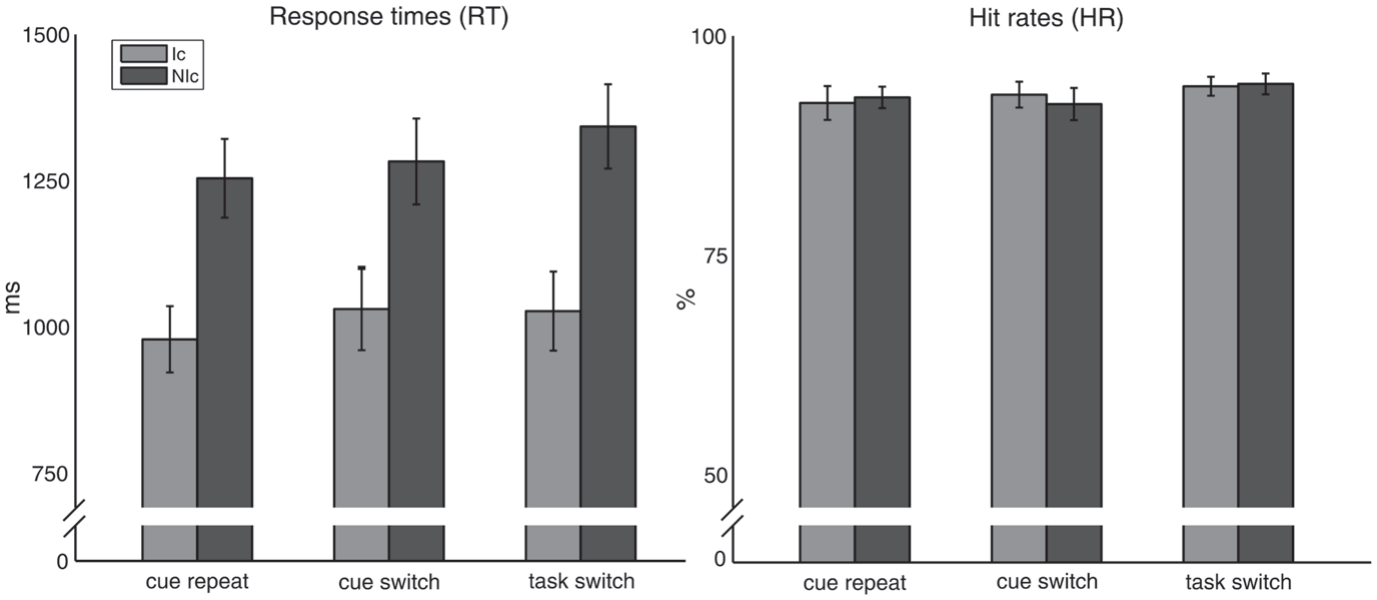
Response times (RT) in milliseconds (ms) and percent hit rates (HR) across the three task conditions for both, Ic trials (light gray) and NIc trials (dark gray). Mean RTs were faster in Ic trials compared to NIc trials, and faster in cue repeat trials compared to task switch trials. No effect on HR was found.

Mean RTs and hit rates were analyzed using 2×3 repeated-measures ANOVAs with factors: Cue type (informative, non-informative) and Task condition (cue repeat, cue switch, task switch). Participants responded faster to informatively cued trials compared to non-informatively cued trials (main effect for Cue: F(1,16) = 224.77, p<0.001; partial η^2^ = 0.9) caused by increased RT for NIc trials (1293 ms) compared with Ic trials (1012 ms). The main effect for Task condition (F(2,32) = 8.41, p = 0.001; partial η^2^ = 0.3) was due to significantly increased RT in trials containing a switch in task (p<0.001, 1185 ms) compared to repeat trials (1116 ms) while the difference between repeat trials and cue switch trials (1157 ms) did not reach statistical significance (p = .067), and no differences were apparent between cue switch and task switch trials (Figure 2). There was no difference in mean RT between cue-switch and task-switch trials, and there was no interaction between the Cue and Task factors (F(2,32) = 2.02; p = 0.15). No significant differences concerning cue or trial type were found on the participants’ hit rates. Noteworthy, hit rates were very high (over 90% across conditions) which confirms that participants managed to correctly implement the corresponding cue-to-response mapping most of the time on task (Figure 2).

### Cue-locked Event-related Potentials

**Figure 3:**
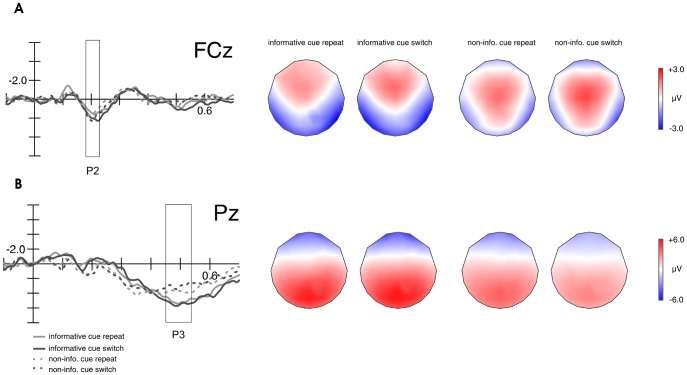
Cue-locked ERPs for the P2 (A) and P3 (B) components for informative and non-informative cue repeat and cue switch trials and their respective topographical distribution.

The hypothesis of whether pure sensory changes, unrelated to any task rule, can modulate behavioral and brain responses to a subsequent target, was addressed through a 2×2×2×3 repeated-measures ANOVA with factors Cue type (informative vs non-informative), Task condition (cue repeat *vs* cue switch), Frontality (either F/FC or CP/P, respectively) and Laterality (left, central, right) on the mean amplitudes of the cue-locked P2 (180–220 ms) and P3 (450–550 ms) components. Figure 3 depicts the results of these analyses. With regard to mean P2 amplitudes, there were no significant main effects or interactions between Cue type and Task condition, and hence, cue switch and cue repeat trials elicited similar P2 amplitudes in informatively and non-informatively cued trials. A main effect for Cue type was found for mean P3 amplitudes (F(1,16) = 22.477; p<0.014; η2 = 0.6), but the interaction between Cue type and Task showed no tendency to significance for this component.

Following up from this first analysis, cue switch and repeat trials were collapsed to focus on the effects of task-relevance on the cue-locked ERPs using a 3×2×3 repeated-measures ANOVA with factors: Trial type (non-informative trials, informative task repeat, informative task switch), Frontality and Laterality as previously described in the Method section. Regarding the P2, this analysis enables us to shed light on the question of whether the P2 is a general change detector or detects task-specific changes. Moreover, modulations in the P3 due to preparatory updating and task anticipation can be investigated.

**Figure 4:**
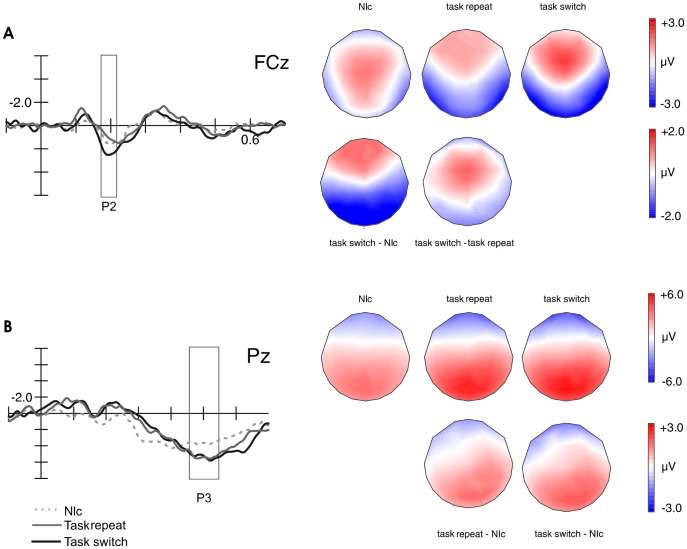
Cue-locked ERPs and topographical distributions for the respective waveforms of the P2 and P3 components. Additionally, topographical maps of difference waveforms are presented. (A) Frontal P2 mean amplitudes were larger for task switch trials compared to task repeat trials and NIc trials. (B) The amplitude of the midparietal P3 component was enhanced for both, informative task repeat and task switch trials compared with NIc trials.

There was a main effect for Trial type (F(2,32) = 6.453, p = 0.004; partial η^2^ = 0.3) for the cue-locked P2 (Figure 4). Post-hoc tests uncovered significantly increased P2 amplitudes in informative task switch trials (2.0 µV) compared to informative task repeat trials (1.1 µV; p = 0.005), and to non-informative cued trials (NIc) trials (1.1 µV; p = 0.007). A main effect for Laterality (F(2,32) = 8.880, p = 0.005, corrected; partial η^2^ = 0.4) uncovered larger mean P2 amplitudes in the left and central electrodes (p = 0.006). The Trial type × Laterality interaction (F(4,64) = 3.589, p = 0.038, corrected; partial η^2^ = 0.2) confirmed the main effect of Trial type (all p<0.02) except for the comparison task switch versus non-informative trials at right electrodes (p = 0.08).

For mean P3 amplitudes (Figure 4), there was a main effect for Trial type F(2, 32) = 9.818, p = 0.00, corrected; partial η^2^ = 0.4). Post-hoc tests revealed that this was caused by significant differences between non-informatively cued trials and informatively cued switch trials (p = 0.005) and compared to informative task repeat trials (p = 0.029).

To further investigate the possible reasons for this absence of differences in mean P3 amplitudes between informatively cued trial conditions, additional ERP analyses were performed. One plausible hypothesis was that a delayed speed of processing of the relatively complex 2-dimensional visual cues might have blurred the expected differences in mean P3 amplitudes (see the Discussion section).

The individual peak P3 latency, as the local positive maximum of the signal within the time window from 400 to 700 ms was obtained automatically for every subject at the same electrode we used for the amplitude analysis, in order to examine whether this measure was affected by Task condition in informatively cued trials. Statistical analysis revealed a main effect for Task (F(2,32) = 5.52, p = 0.009; partial η^2^ = 0.3). Post-hoc comparisons uncovered significantly delayed peak P3 latencies for switch (539 ms) compared to repeat trials (503 ms; p = 0.008). No significant differences were found for cue switch trials (514 ms) compared to any other task condition.

### Target-locked Event-related Potentials

**Figure 5:**
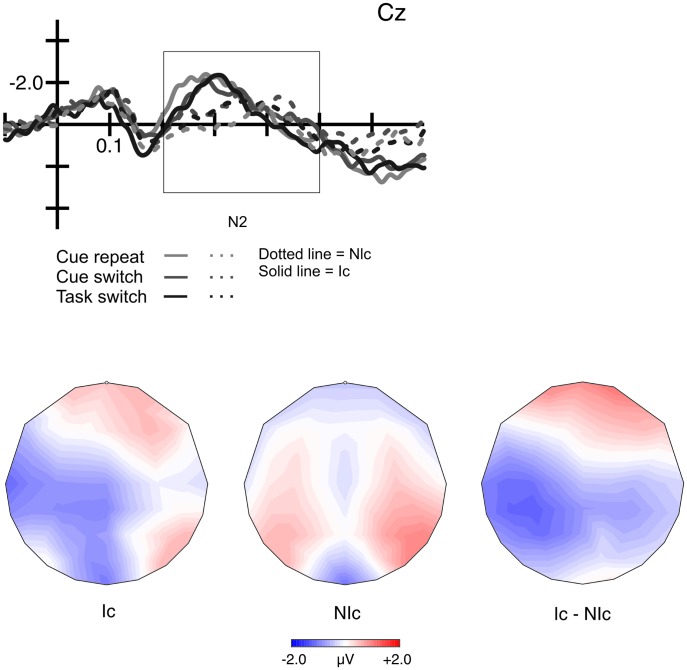
Target-locked ERPs and topographical distributions for the informatively and non-informatively cued waveforms in the N2 time window. Moreover, the topographical map of their difference waveform is shown. Amplitudes were enhanced for Ic trials compared to NIc trials for both ERP components. Shortest N2 peak latencies occurred in repeat trials.

The same repeated-measures ANOVA design used in the behavioral analyses was also used with target-locked ERP data, including the factors Cue type (informative, non-informative), Task condition (cue-repeat, cue-switch, and task-switch) and Laterality (left, central, right). Repeated measures ANOVAs were performed on the target-locked N2. ERP results for the target-locked components are displayed in Figure 5.

The analysis of N2 peak amplitude revealed a main effect for Cue type with amplitudes being more negative for informative trials (F(1.16) = 8.11, p = 0.012; partial η^2^ = 0.3). As for the latency we also found a main effect for Laterality (F(2,32) = 7.8, p = 0.002) due to highest amplitudes at Cz. For the N2 peak latency, a main effect for Task condition was found (F(2,32) = 3.5, p = 0.042; partial η^2^ = 0.18), which was caused by longer N2 latencies for cue switch compared to cue repeat trials (328 ms versus 307 ms; p = 0.042). Task switch trials did not yield different N2 latencies (321 ms) compared to the other two task conditions. A main effect for Laterality was due to shorter peak N2 latencies at Cz compared to C2 compared to C1.

## Discussion

The current study aimed to elucidate processes related to anticipatory task-set updating during the foreperiod of a task-cueing paradigm considering the combined influence of changes in sensory cues and abstract task rules, as well as the extraction of informational content (foreknowledge) in anticipation of target onset. RT was modulated by the cue type and task condition without an interaction between these factors. The amplitude of an endogenous P2 component was enhanced for informatively cued task switch trials suggesting a considerably fast process of contextual task-relevant “change detection”. The cue-locked P3 amplitude was enhanced for all informatively cued trials, probably reflecting preparatory updating for informative trials. This process was prolonged in informative switch trials compared to repeat trials as indicated by prolonged P3 latencies. Sharper target-locked N2 amplitudes for informatively cued trials suggested better task-implementation and prepared responses. Shorter N2 latencies for all repeat compared to task switch trials were related to faster response mapping.

### Performance Data

In line with former work the present study revealed faster RTs in informatively cued trials compared to non-informatively cued trials [4], [9]. As expected, the slowest RTs were found in switch trials, while participants responded faster in repeat trials in the present study replicating the typical switch costs [3]–[5], [10], [11], [15], [23]. As in previous studies, no interaction was found between the two factors [4], [9]. Trials containing a cue switch prolonged response times, thus confirming that sensory updating in the visual modality can affect response times in a similar way as auditory changes do, though this effect was weak and the difference marginally failed significance [15], [17], [21]. Importantly, former work showed different sensibility to this “distraction” depending on individual variability in the DAT1 gene for the dopamine transporter and subject variability was indeed high in cue-shift informative trials in the present study [21]. In contrast to former studies, hit rates were not affected by foreknowledge or trial type [4], [19], [21]. A possible reason for these null hit rate effects could be the very high overall accuracy of participants in our study (with a mean hit rate of over 90% correct responses).

### The Relevance of Pure Sensory Cue Changes

There was a difference between mean P3 amplitudes for informatively and non-informatively cued trials. However, no differences in the cue-locked P2 and P3 components could be found between task conditions. Mean P2 amplitudes were larger for cue switch compared to cue repeat trials but this difference failed significance. This null result fails to replicate the cue switch effect found in previous studies [13], [15], [18], [19], [21], [29]. Importantly, it should be noted that most previous studies did not isolate cue switching effects from the anticipatory task-relevant information about the upcoming task [13], [14]. Consequently, a direct analysis of both, Cue type and cue switch effects has never been done before. The present P3 data suggest that task information is most relevant in order to prepare a response mapping. Contrariwise, the influence of bottom-up processes seems to be very little. The information of the cue, the top-down processing, is important. This is also supported by a more frontal and more focused P2 distribution for informative compared to non-informative trials as visible in Figure 3. Hence the present results can help clarify the ongoing discussion on how much of the task switch cost is related to a pure sensory change, and suggest that simple perceptual cue repetition might not be sufficient to explain the benefits found for informative repeat trials, as other task preparation processes seem to be involved. Admittedly, this null effect could also be due to the weaker cue switch effects obtained with our visual displays, as compared with the auditory cues used by previous studies [15], [19], [21]. Future research should be done to further explore the relative impact of pure sensory cue changes on behavioral switch costs independent from task relevance in informative versus non-informative cues.

The present study further explored whether cue repetitions, that are often held responsible for the cost benefit in task repeat trials, could also depend on task-relevant information. In addressing this question, a 2∶1 cue:task mapping was used in order to control for cue switch costs as opposed to task switch costs in both informative and non-informative trial conditions [13], [18]. Though transitional cues have been used in previous studies [17], [19], our paradigm offers the advantage of a randomized order of both cue and task manipulations. However, this would not be the case if using transitional cues as non-informatively cued switch trials could only occur after an informatively cued trial but never following a non-informatively cued trial (as this order allows a cue repeat only). Moreover, previous contradictory results regarding the effect of cue switches might be due to their different probability of occurrence [13], [14]. Like recent ERP studies we used the 2∶1 cue:task mapping since ERP components such as the P3 are sensible to stimulus probability [18], [21], [41].

### Parallel versus Serial Cue and Task Processing

The fast detection of task-relevant cue changes was indexed by the P2 component. Increased frontal P2 amplitudes were elicited by informatively cued task switch trials compared to informatively cued task repeat trials, or non-informatively cued trials. The sensitivity of the frontal cue-locked P2 component to anticipatory task-set updating (in repeat versus switch trials) replicates previous work [23], [29]. Moreover, these results allow us to complement the common interpretation of the frontally distributed P2 component in terms of a general “change detector”, since this component does not only reflect task-set updating in switch trials, but is also modulated in anticipation of task-relevant information [22], [23]. This is supported by the fact that task switch trials differ significantly from task repeat trials and from non-informative trials. Taken together, these results lead us to the interpretation that the cue-locked P2 component conveys task-relevance, and hence, it could be regarded as a task-specific “change detector”. This result is important as it indicates that even as early as during initial cue processing, bottom-up sensory aspects are not necessarily processed before top-down task relevant aspects [27]. Possible influences for cue changes on the P2 could be addressed in a future study using auditory cues as they seem to elicit stronger effects. Probably, the frontal P2 is related to task-set activation and cue-retrieval processes as suggested previously [25]. Likewise, increased P2 amplitudes have been reported for trials or tasks including preparatory control and stimulus evaluation [11], [23], [29].

With the present study we show that the frontal P2 component is sensitive to these two distinct but related processes of anticipatory task-set retrieval or activation on the one hand, and with the interruption, inhibition, and deactivation of a previously active task set on the other hand. In a second step the processes of cue-response mapping and the reloading/updating of stimulus-response mappings take place as mirrored in the cue-locked P3 component. Increased mean P3 amplitudes for informatively cued cue-switch and a task-switch trials compared to non-informatively cued trials, replicates former studies and goes in line with the sensitivity of the cue-locked P3 component regarding preparatory control of task-set switching [4], [15], [18], [25], [42]. The delayed P3 peak latencies for switch compared to repeat cues mirrored a prolonged stimulus evaluation process, and thus, may have been putatively associated with delayed task-set shifting given our complex task switch cues [43]–[45]. On the other hand, a cue switch did not prolong P3 latencies in the present study.

However, the present results did not show the typical enhancement of cue-locked P3 amplitudes in response to task switch cues compared to task repeat cues, as shown in most previous studies, although our results are consistent with the strong effects of foreknowledge in behavioral performance [15], [19], [21]. The null result regarding task-switching effects in the P3 amplitudes might be due to the complex cue design as it has been argued in previous studies that the encoding of complex cues might be a “task” itself [3]. The observed P3 latency differences lend support to such a hypothesis. The readout of our complex 2-dimensional visual cues may have induced a significant delay compared to simpler visual and auditory cues. In order to examine this idea more directly, we analyzed peak P3 latencies for informative trials and also conducted a new behavioral follow-up study. In this new study, we re-defined the meaning of informative cues so that a horizontal rectangle now instructed the color rule, and a vertical rectangle now instructed the shape rule, independently from the orientation of the stripes within those rectangles. This “easy version” of our task-cueing protocol was then run on four participants from the original sample, plus another five new subjects who performed both the new and old versions of our task-switching protocol. The order of both versions was counterbalanced between subjects. Taken together, nine healthy individuals (2 male, mean age = 27 years ±1.23 SEM, range = 21–33 years) participated in this follow-up study. As predicted by our cue complexity hypothesis, mean RTs in the easy version were significantly faster for cue-repeat (p = 0.004) and cue-switch trials (p = 0.009) compared to the same conditions in the original task-cueing protocol (960 ms compared to 1038 ms, and 989 ms compared to 1059 ms, respectively), as indicated by a significant interaction between Cue complexity x Task condition (F(2,16) = 4.639; p = 0.026). Taken together, the re-analysis of cue-locked peak P3 amplitude and latency as well as the behavioral results of the new follow-up study suggest that the information read-out of complex visual cues in the original task-cueing protocol became a task in itself as suggested by Monsell [3].

### Preparation for the Upcoming Task

Mean N2 amplitudes were enhanced for informatively cued trials but were clearly diminished –or even abolished– in non-informatively cued trials. The N2 has been related to the monitoring of action which in turn has been related to the function of the anterior cingulate cortex (ACC) [33], [34]. Regarding the task-switching literature, there have been inconsistent results for N2 effects; some studies found enhanced N2 amplitudes for switch trials compared to repeat trials while others found the reversed pattern or no effects at all (for an over view see [32]). Gajewski and colleagues interpreted N2 modulations as being related to stimulus classification during target-selection and decision processes. Consequently, the sharper N2 peak amplitudes in informatively cued trials could reflect anticipatory task preparation [28], [38]. There is also evidence that the N2 itself is mirroring a general process of selection (modulated by interference) and cognitive control over selective attention [31], [32]. For the current study, the observed enhancement of target-locked N2 amplitudes for informatively cued trials might mirror top down control in terms of implementation the current task rule, and the selection of the appropriate correct response button for a particular target stimulus [31], [32]. However, the present data cannot confirm any task-switch effect in the N2 amplitude.

In contrast to peak amplitudes, N2 peak latencies were modulated by task condition. Statistical analyses revealed that cued repeat trials lead to shorter latencies compared to switch trials. A former study linked N2 latency differences with the time participants need to achieve a certain level of categorization of stimuli [46]. For our data this means that the monitoring processes occur earlier in time for repeat trials, and consequently, the stimulus-response remapping may be addressed faster than for switch trials. This goes in line with results by Swainson and colleagues, and with the shorter RT for repeat trials in both cue conditions [47]. Likewise, it agrees with the idea that anticipatory preparation cannot fully overcome task switching costs [4], [7]. We expected to find differences between cue repeat and task switch trials. Instead, N2 latencies for task switch trials were delayed compared to cue repeat trials and faster than for cue switch trials. This suggests that stimulus-response re-mapping maybe prolonged in trials where a cue-switch is not associated with a switch in task. One explanation for this result might be that participants seem to generate expectancies regarding the probabilities of the upcoming choice if the inter-trial interval is not particularly short, and thereby tend to overestimate the probability of a switch [48]. A conflict occurs as the sensory switch is strengthening this expectation of a switch by bottom-up processes but no task-switch is required later. There is an “incongruent cue-task transition” whenever a cue switch is not accompanied by a switch in task [29].

Regarding our third research question, how the benefit for task preparation will affect target-locked brain responses, we can show that due to informative cues and task-preparation in the CTI response mapping can be accomplished more efficiently after target-onset but is faster in all repeat trials. Importantly, the current study could not find an interaction between the Cue and Task factors. Consequently, we did not find any bias of a non-informative cue switch for the task preparation. As in the cue-locked ERPs, a non-informative cue switch seems to elicit a mere sensory effect but do not affect task related processing stages. Apparently, there seems to be an undue switch preparation in all cue switch trials that can be inferred from the prolonged N2 latencies for these trials.

### Conclusion

With the present study we investigated three different questions regarding the extraction of cue information and its importance for task-switching processes: (1) Does a sensory change in a cue that is unrelated to the upcoming task modulate performance and brain activity even if this sensory change does not carry information about the upcoming task? (2) Can we find indisputable evidence to support a serial or parallel processing of cue and task information? (3) How does the purported benefit of anticipatory task preparation affect target processing and the target-locked ERPs? The present data suggest that sensory cue changes do affect cue-locked ERPs only when they convey contextual information about upcoming task performance. This is an important result for the interpretation of cue switch costs. Our data shows that differences in ERP components between cue repeat and cue switch trials are not related to a mere sensory effect but rather reflect a task related process. We found no evidence to support a serial or parallel processing of cue and task information but rather fast task-relevant change detection (P2). The following process of cue-response mapping and the reloading/updating of stimulus-response mappings occur later (P3). Importantly, our data revealed that task-relevant change detection occurs quite early (starting from 180 ms). Moreover, it highlights the importance of the P2 for the processing of cognitive processes. Finally, task preparation is generally advantageous for response re-mapping after target-onset (N2 amplitude) though task monitoring is fastest in repeat trials (N2 latency). Noteworthy, a non-informative cue switch seems not to affect task preparation in any particular way.
